# Small Intestine and Vermiform Appendix Lengths in Healthy Adults: Intraoperative Morphometric Data from a Living Donor Cohort

**DOI:** 10.3390/jcm15051747

**Published:** 2026-02-25

**Authors:** Emrah Sahin, Adem Tuncer, Cuneyt Kayaalp, Abuzer Dirican, Bulent Unal, Servet Karagul

**Affiliations:** 1 Department of General Surgery, Istanbul Aydin University, 34295 Istanbul, Turkey; ademtuncer89@hotmail.com (A.T.); abuzerdirican@hotmail.com (A.D.); ddozelsaglik@gmail.com (B.U.); 2 Department of General Surgery, Istanbul Atlas University, 34408 Istanbul, Turkey; cuneytkayaalp@gmail.com (C.K.); servetkaragul@hotmail.com (S.K.)

**Keywords:** appendix, intestine, small, living donors, organ size, reference values, anatomy

## Abstract

**Background/Objectives**: Small intestine and vermiform appendix lengths are critical anatomical parameters with significant implications for bariatric surgery, transplantation, and the management of short bowel syndrome. However, reliable normative data derived from direct in vivo measurements in healthy individuals remain limited. This study aimed to establish reference values in a homogeneous cohort of healthy living liver donors and to investigate the potential anatomical and functional correlation between these two structures based on their shared embryological origin and immunological roles. **Methods**: Data from 99 healthy living liver donors representing a multi-ethnic population were analyzed. Intraoperative measurements were performed using a standardized surgical technique: small intestine length was measured from the ligament of Treitz to the ileocecal valve, and appendix length from the base to the tip. Relationships between morphometric measurements and anthropometric variables were evaluated. **Results**: The mean small intestine length was 630 ± 104.7 cm, and the mean appendix length was 84.8 ± 21.1 mm. Small intestine length was significantly greater in males (*p* = 0.011), while appendix length showed no sex-based difference. A weak but statistically significant positive correlation was found between small intestine length and appendix length (*r* = 0.237; *p* = 0.021). Furthermore, an appendix length ≥ 8 cm was associated with a higher probability of having a small intestine length > 600 cm. Notably, the retrocecal appendix position was observed in only 4.0% of donors, a prevalence substantially lower than that reported in appendicitis series, suggesting it may be a risk factor for inflammation. **Conclusions**: This study reports descriptive measurements of small intestine and appendix lengths in healthy individuals undergoing donor hepatectomy. The relationship between the appendix and the small intestine appeared weak and should be interpreted as exploratory, warranting further investigation without implications for clinical decision-making.

## 1. Introduction

The length of the small intestine is a fundamental anatomical variable that plays a decisive role in digestive physiology and surgical outcomes. Although classical anatomical studies have attempted to define “normal” values, results have varied widely due to methodological differences, such as the use of cadavers affected by postmortem tissue relaxation [1,2]. Consequently, establishing reliable normative reference ranges based on living, healthy individuals remains a challenge in surgical literature.

From a clinical perspective, knowing the precise length of the small intestine is critical, particularly in the era of metabolic and transplant surgery. In short bowel syndrome, the residual intestinal length is the strongest predictor of nutritional independence; patients with less than 200 cm of functional bowel face a high risk of chronic malabsorption [3]. Similarly, in bariatric procedures such as Roux-en-Y gastric bypass, standardizing the length of the alimentary and biliopancreatic limbs assumes a relative uniformity in total bowel length [4]. However, if total length varies significantly among individuals, a fixed-length bypass could lead to insufficient weight loss in patients with long intestines or severe malnutrition in those with short intestines [5]. Therefore, preoperative estimation or reliable population norms are essential for tailoring surgical approaches.

The vermiform appendix, traditionally regarded as a vestigial organ, is now recognized as a specialized component of the gut-associated lymphoid tissue (GALT) system. It serves as a reservoir for commensal bacteria and plays a role in mucosal immunity. Its length, diameter, and anatomical variations may directly influence both the clinical presentation and the surgical approach [6,7]. Despite their distinct morphologies, the small intestine and the appendix share a common embryological origin from the midgut. Biologically, it is plausible to hypothesize a functional “anatomical continuum” between these structures; a longer small intestine, possessing a larger mucosal surface area and microbial load, might require a more developed lymphoid organ—i.e., a longer appendix—for adequate immunoregulatory support [8,9]. However, this potential morphometric relationship has not been systematically evaluated in healthy human populations.

Most existing data on these organs are derived from cadaveric studies or patient cohorts undergoing surgery for specific pathologies, which may introduce bias due to inflammation or anatomical distortion [10,11]. Intraoperative measurements performed in living patients typically involve heterogeneous groups. Therefore, reliable normative anatomical data derived from a homogeneous cohort of completely healthy individuals, obtained using direct intraoperative measurements—the accepted gold standard—are still needed [12].

The primary aim of this study was to establish normative reference values for small intestine and appendix lengths using direct intraoperative measurements in a multi-ethnic cohort of healthy living donors. Secondly, we aimed to test the hypothesis that appendix length is positively correlated with small intestine length, potentially serving as a surrogate marker in surgical planning.

## 2. Materials and Methods

### 2.1. Study Design and Ethical Approval

This single-center, descriptive, retrospective cohort study was conducted at the Department of General Surgery, Istanbul Aydın University. The study protocol adhered strictly to the ethical principles of the Declaration of Helsinki and received formal approval from the Istanbul Aydın University Non-Interventional Clinical Research Ethics Committee on 3 April 2024 (Approval No. 29/2024). To ensure patient confidentiality, all data were fully anonymized prior to analysis.

### 2.2. Participant Selection

The study population consisted of healthy individuals who underwent living-donor liver transplantation surgery between October 2023 and July 2025. A total of 103 consecutive donor candidates were initially assessed. Rigorous exclusion criteria were applied to ensure a homogeneous “healthy” cohort: individuals with a prior history of appendectomy (*n* = 1), cases disqualified from donation due to intraoperative findings (*n* = 1), and donors with incomplete morphometric data (*n* = 2) were excluded. Consequently, the final analysis included 99 healthy living liver donors.

Inclusion criteria were defined as: age between 18 and 55 years, undergoing surgery as a living liver donor, no history of major abdominal surgery, and absence of known intestinal pathology. Candidates for living liver donation undergo an extensive preoperative screening process to confirm optimal physiological health, making this cohort a unique representation of the “normal” human population, free from the biases often found in cadaveric or symptomatic patient series.

### 2.3. Data Collection and Measurement Techniques

Demographic and anthropometric data, including age, sex, height (cm), weight (kg), and body mass index (BMI), were recorded. Standardized Intraoperative Measurement: All measurements were performed by the same transplant surgeon and the same observer surgeon to eliminate inter-observer variability. Measurements were taken intraoperatively under general anesthesia, ensuring complete muscle relaxation.

- Small Intestine Length (SIL): Previous studies in the literature have found that bowel length changes with contraction in repeated measurements [13]. Based on this information, we recorded the patient’s small bowel length by taking a single measurement as the first procedure after laparotomy, without performing any intra-abdominal manipulation. The length was measured from the ligament of Treitz to the ileocecal valve along the antimesenteric border. To avoid the confounding effect of tissue elasticity, a standardized technique was used where the bowel was measured segment by segment using a 70 cm sterile nylon surgical tape, without applying traction and measurement of bowel length was not repeated. This technique aligns with established methods recognized in the literature as the most accurate for in vivo assessment [12,13,14].
- Appendix Length: The vermiform appendix was measured from its base at the cecal wall to its distal tip. The free segment was gently extended to its physiological length without overstretching. Measurements were taken only when the organ was in a relaxed state, avoiding spasm.
- Ethical Consideration for Retrocecal Appendix: In cases where the appendix was located in a retrocecal position, it was measured in situ. Mobilization of a healthy retrocecal appendix solely for measurement purposes was avoided to prevent unnecessary tissue trauma and potential postoperative complications for the healthy donor.

Anatomical variations, such as Meckel’s diverticulum, were also documented during the exploration.

### 2.4. Statistical Analysis

Data were entered into Microsoft Excel 365 (Microsoft Corp., Redmond, WA, USA) and analyzed using SPSS Statistics for Windows, version 25.0 (IBM Corp., Armonk, NY, USA). The normality of continuous variables was assessed using the Kolmogorov–Smirnov test and visual inspection of histograms. Continuous variables with a normal distribution were presented as mean ± standard deviation (SD), while those with a non-normal distribution were expressed as median (minimum–maximum). Categorical variables were summarized as frequencies (*n*) and percentages (%).

For comparisons between two independent groups (e.g., male vs. female), Student’s *t*-test was used for normally distributed data, and the Mann–Whitney U test for non-normally distributed data. The relationships between morphometric measurements (SIL, appendix length) and anthropometric variables were evaluated using Pearson correlation analysis.

The sample size was not determined by an a priori power analysis; rather, it included all consecutive eligible cases within the study period. Therefore, the study was designed as a hypothesis-generating exploratory analysis. Cut-off values for appendix length and subsequent ROC (Receiver Operating Characteristic) analyses were conducted as part of this exploratory approach to investigate the predictive value of appendix length for intestinal length. A *p*-value of <0.05 was considered statistically significant.

## 3. Results

### 3.1. Demographic and Anthropometric Characteristics

The study cohort consisted of 99 healthy living liver donors, comprising 46 females (46.5%) and 53 males (53.5%). The mean age was 33.6 ± 9.6 years (range: 18–55 years). The participants represented a physically healthy population with a mean BMI of 24.0 ± 3.5 kg/m^2^. Detailed baseline characteristics are presented in Table 1.

### 3.2. Small Intestine and Appendix Morphometry

The mean small intestine length (SIL) for the entire cohort was 630 ± 104.7 cm, with a wide range of variation from 432 cm to 910 cm. A statistically significant difference was observed between sexes; males had a longer mean SIL compared to females (654.8 ± 110.2 cm vs. 601.5 ± 91.1 cm; *p* = 0.011).

The mean appendix length was 84.8 ± 21.1 mm (range: 50–150 mm). Unlike the small intestine, appendix length did not differ significantly between males (86.5 ± 21.0 mm) and females (82.8 ± 21.4 mm) (*p* = 0.389). The distribution of measurements by sex is summarized in Table 2.

### 3.3. Geographical Distribution of the Cohort

A unique strength of this study is the multi-ethnic composition of the donor pool, representing 13 different countries. While the majority of donors were of Turkish origin (60.6%), a significant portion (39.4%) consisted of international donors, predominantly from North African and Middle Eastern regions. Subgroup analysis of nationalities with *n* ≥ 5 revealed that mean SIL values were relatively consistent across different ethnic groups, ranging from 622 cm (Iraq) to 643 cm (Algeria). The geographical distribution and corresponding SIL values are detailed in Table 3.

### 3.4. Anatomical Variations

Anatomical variations were meticulously documented during the surgical exploration. A retrocecal appendix position was identified in only 4 donors (4.0%). This prevalence is notably lower than rates frequently reported in acute appendicitis series. Additionally, Meckel’s diverticulum was incidental detected in 2 donors (2.0%). No other significant intestinal anomalies were observed in this healthy cohort.

### 3.5. Correlation Analysis

Pearson correlation analysis demonstrated that SIL was positively correlated with both height (*r* = 0.256, *p* = 0.011) and weight (*r* = 0.261, *p* = 0.009). However, no significant correlation was found between SIL and age (*p* = 0.927), suggesting that intestinal length remains stable in adulthood. All correlation coefficients and significance levels are summarized in Table 4.

**Figure 1:**
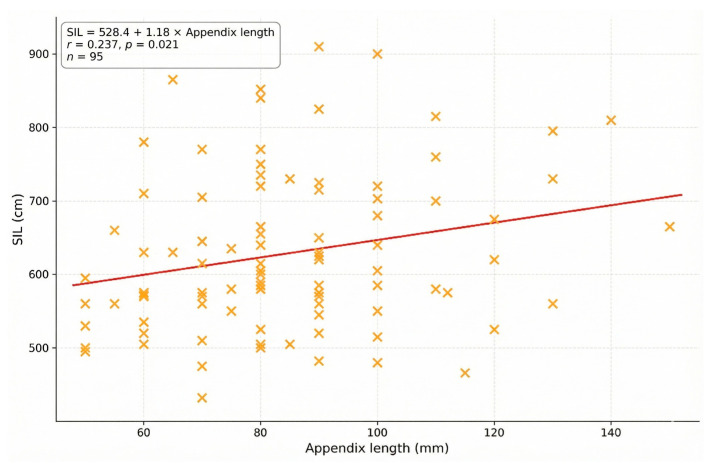
Correlation between small intestine length (SIL) and appendix length (*n* = 95). The linear regression equation (indicated by the red line) is: SIL = 528.4 + 1.18 × appendix length (mm). The scatter plot demonstrates a weak but statistically significant positive correlation (*r* = 0.237, *p* = 0.021). Four donors with a retrocecal appendix were excluded from the correlation analysis due to inability to obtain standardized measurements. The wide dispersion reflects the multifactorial nature of intestinal length determination.

A key finding of this study was the statistically significant positive correlation between small intestine length and appendix length (*r* = 0.237; *p* = 0.021). As illustrated in Figure 1, individuals with longer appendices tended to have longer small intestines, supporting a weak association between small intestine length and appendix length.

### 3.6. Predictive Value of Appendix Length

To evaluate the clinical utility of appendix length as a predictor for intestinal length, donors were stratified based on appendix length. Donors with an appendix length ≥ 8 cm had a significantly longer mean SIL compared to those with an appendix < 8 cm (645.4 ± 107.6 cm vs. 595.6 ± 94.0 cm; *p* = 0.021). Detailed comparative statistics for these subgroups are provided in Table 5.

**Figure 2:**
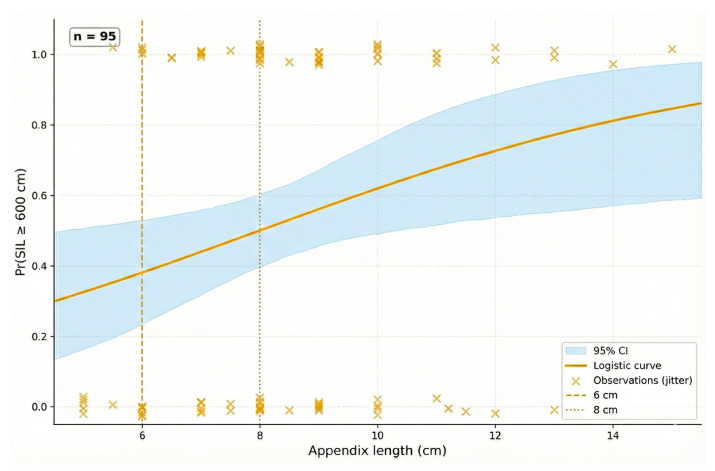
Logistic regression curve showing the predicted probability of having a small intestine length (SIL) ≥ 600 cm as a function of appendix length (*n* = 95). The solid line represents the estimated probability derived from the logistic model (OR = 1.28 per 1 cm increase; 95% CI: 1.03–1.58; *p* = 0.025). The light blue shaded area represents the bootstrap-derived 95% confidence interval of the predicted probabilities. Individual observations are displayed as jittered points at 0 (SIL < 600 cm) and 1 (SIL ≥ 600 cm). The dashed vertical line indicates the 6 cm threshold and the dotted vertical line indicates the 8 cm threshold.

Further exploratory analysis using logistic regression indicated that for every 1 cm increase in appendix length, the likelihood of having a small intestine longer than 600 cm increased by a factor of 1.28 (OR = 1.28; 95% CI: 1.03–1.58; *p* = 0.025). The logistic regression model demonstrating this probability is shown in Figure 2.

**Figure 3:**
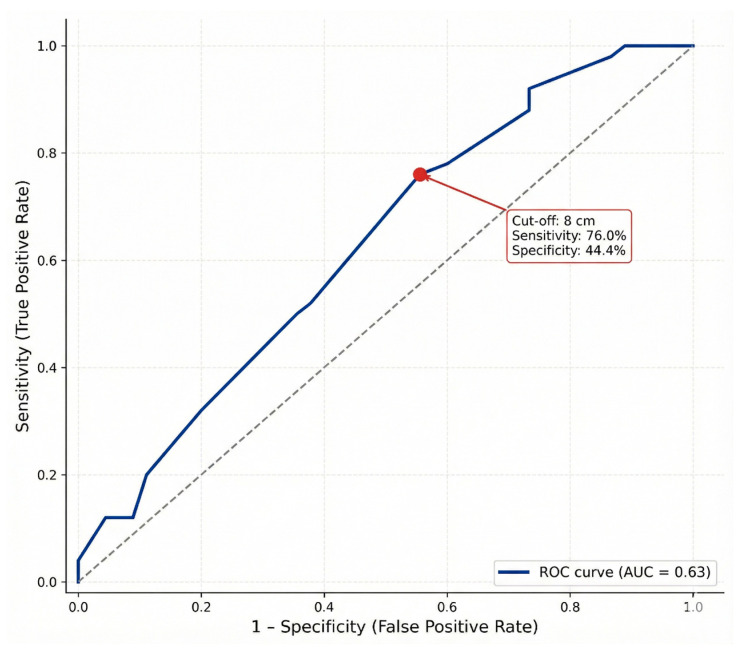
Receiver Operating Characteristic (ROC) curve illustrating the discriminatory ability of appendix length in predicting a small intestine length ≥ 600 cm (*n* = 95). The Area Under the Curve (AUC) was 0.63 (95% CI: 0.51–0.74). At the optimal cut-off of 8.0 cm, appendix length yielded a sensitivity of 76.0% and a specificity of 44.4%. The red dot on the curve indicates the operating point corresponding to this threshold. The grey dotted line represents the line of no discrimination (random chance).

Finally, to define a practical cut-off value for surgical decision-making, a Receiver Operating Characteristic (ROC) curve analysis was performed. The analysis confirmed the discriminatory ability of appendix length in predicting a small intestine length ≥ 600 cm, yielding an Area Under the Curve (AUC) of 0.63 (95% CI: 0.51–0.74). The overall predictive power was moderate and the analysis identified 8.0 cm as the optimal threshold. At this cut-off point, appendix length demonstrated a sensitivity of 76.0% and a specificity of 44.4%, suggesting that donors with an appendix shorter than 8 cm are less likely to have a long small intestine. The ROC curve illustrating this diagnostic performance is presented in Figure 3.

## 4. Discussion

This study contributes to the surgical literature by providing normative reference values for small intestine and vermiform appendix lengths obtained through direct intraoperative assessment in a strictly screened, healthy, and geographically diverse cohort of living liver donors. The geographical analysis is descriptive and not intended to demonstrate true inter-population differences. By focusing exclusively on healthy individuals, we aimed to eliminate the confounding biases often introduced by inflammation, chronic disease, or postmortem tissue changes that have limited the accuracy of previous anatomical studies.

The mean small intestine length observed in our cohort was 630.0 ± 104.7 cm. This value represents a physiological middle ground between the longer lengths frequently reported in cadaveric studies and the shorter lengths seen in some living patient series. These variations are likely attributable to differences in measurement standardization, as well as anthropometric and genetic differences among populations. A detailed comparison of our findings with previous cadaveric and in vivo studies is presented in Table 6.

For instance, Ref. [1] reported a mean length of roughly 690 cm in cadavers, a discrepancy likely attributable to the postmortem loss of smooth muscle tone leading to tissue elongation. Conversely, living patient cohorts often yield lower means, such as the ~460 cm reported by [15] or 506 cm by [16]. Our finding of 630 cm suggests that in a healthy individual under general anesthesia, where muscle tone is preserved but relaxed, the measurements reflect the most accurate “functional” anatomical length. Recent studies on bariatric populations have also highlighted the importance of these measurements for predicting malnutrition risks [17].

**Table 6:** Comparison of mean small intestine length (SIL) reported in selected studies versus the current study.

Study	*n*	Population	Measurement Method	Mean SIL ± SD (Range) (cm)
Hounnou et al. [1]	200	Cadaver	Anatomical	690 ± 129 (470–970)
Gondolesi et al. [10]	20	Deceased Donor (Adult)	Intraoperative	356.0 ± 58.3
Teitelbaum et al. [16]	240	Living patient (USA)	Intraoperative	506 ± 105 (285–845)
Hosseinpour et al. [15]	100	Living patient (Iran)	Intraoperative	459.6 ± 78.47 (285–620)
Sayadishahraki et al. [17]	150	Bariatric patient (Iran)	Intraoperative	545 ± 162.68 (205–900)
Current Study	99	Healthy Donor (Multi-ethnic)	Intraoperative	630 ± 104.7 (432–910)

Note: This table highlights the variability in reported intestinal lengths based on the population type (cadaver vs. living) and measurement technique. Abbreviation: SIL: Small Intestine Length.

Regarding anthropometric variables, our data confirmed that males possess significantly longer small intestines than females (654.8 cm vs. 601.5 cm; *p* = 0.011), likely due to greater body surface area and metabolic mass. While some meta-analyses have suggested varying relationships between appendiceal morphometry and body dimensions [18], our findings align with studies indicating a positive correlation between organ size and height/weight [19]. The absence of a correlation with age supports the view that intestinal length remains anatomically stable in adulthood [2].

Beyond standard morphometry, this study offers novel insights into the biological relationship between the appendix and the small intestine. We identified a weak positive correlation between the lengths of these two organs (*r* = 0.237; *p* = 0.021), but it is not yet possible to draw definitive conclusions. The predictive value is exploratory and appendix length can not currently guide surgical decision-making. This emphasizes the need for further large-scale, well-designed prospective studies to confirm these findings. Since both structures share a common embryological origin from the midgut and are integral components of the GALT system, this correlation is biologically plausible [7]. It is reasonable to postulate that a longer small intestine, which presents a larger mucosal surface area and sustains a higher microbial load, requires a more developed lymphoid reservoir for immune regulation [8,9]. This functional linkage is further supported by animal models where compensatory hypertrophy of the appendix—or cecal lymphoid patches—has been observed following massive small-intestinal resection [20].

These anatomical variations have profound implications for modern surgical practice. Procedures such as Roux-en-Y gastric bypass often employ standardized limb lengths under the assumption of anatomical uniformity. However, our data reveal a massive range in total intestinal length (432–910 cm). Bypassing 150 cm in a patient with a 432 cm total length represents a critical reduction in absorptive capacity, whereas the same bypass in a patient with 910 cm constitutes a much smaller fraction [4,5]. Similarly, in the management of short bowel syndrome, where the residual length is the strongest predictor of nutritional autonomy [3], our predictive analysis suggests that appendix length could serve as a surrogate marker.

Perhaps the most striking incidental finding of our study was the remarkably low prevalence of the retrocecal appendix position (4.0%) in our healthy donors. This stands in stark contrast to the 25–79% prevalence frequently reported in acute appendicitis series [21,22]. Since our cohort consists of healthy individuals screened to exclude pathology, this discrepancy implies that the retrocecal position can be a risk factor for appendicitis. The observed prevalence differs from that reported in inflamed appendix cohorts, and this observation seems worthy of further investigation in new studies. The anatomical configuration may predispose the organ to kinking, impaired lymphatic drainage, or luminal stasis, thereby increasing susceptibility to inflammation [23]. Similarly, the 2.0% prevalence of incidental Meckel’s diverticulum aligns with general population rates reported in systematic reviews [24,25], further supporting the representativeness of our cohort.

## Figures and Tables

**Table 1:** Demographic and anthropometric characteristics of the donors (*n* = 99).

Parameter	Mean ± SD/*n* (%)	Range (Min–Max)
Age (years)	33.6 ± 9.6	18–55
Sex	Female 46 (46.5%) Male 53 (53.5%)	–
Height (cm)	170.7 ± 9.6	147–192
Weight (kg)	70.1 ± 12.8	46–106
BMI (kg/m^2^)	24.0 ± 3.5	16.5–34.4

Note: Data are reported as mean ± standard deviation and range (minimum–maximum). Abbreviation: BMI: Body Mass Index.

**Table 2:** Small intestine and appendix length measurements by sex.

Parameter	Entire Cohort	Female	Male	*p* Value *
SIL (cm)	630.0 ± 104.7	601.5 ± 91.1	654.8 ± 110.2	**0.011**
Appendix (mm)	84.8 ± 21.1	82.8 ± 21.4	86.5 ± 21.0	0.389

Note: Data are presented as mean ± standard deviation. * Differences between groups were evaluated using Student’s *t*-test. Abbreviation: SIL: Small Intestine Length.

**Table 3:** Distribution of Small Intestine Length (SIL) by Nationality (Groups with *n* ≥ 5).

Nationality	*n* (%)	Mean SIL ± SD (cm)	Range (Min–Max)
Turkey	60 (60.6%)	623.5 ± 100.8	432–865
Algeria	13 (13.1%)	643.6 ± 101.5	482–795
Morocco	8 (8.1%)	635.4 ± 83.9	525–720
Iraq	6 (6.1%)	622.0 ± 125.8	475–852
Others *	12 (12.1%)	638.1 ± 112.4	466–910

Note: * Includes donors from 9 other countries with *n* < 5. Data are presented as mean ± standard deviation and range (minimum–maximum). This distribution highlights the multi-ethnic composition of the study cohort. Abbreviation: SIL: Small Intestine Length.

**Table 4:** Correlation coefficients between morphometric and anthropometric variables.

Parameter	Height	BMI	Age
SIL	0.256 (*p* = 0.011)	0.261 (*p* = 0.009)	−0.009 (*p* = 0.927)
Appendix	0.214 (*p* = 0.037)	0.292 (*p* = 0.004)	−0.072 (*p* = 0.486)

Note: Data represent Pearson correlation coefficients (*r*) and corresponding *p*-values. Abbreviations: SIL: Small Intestine Length; BMI: Body Mass Index.

**Table 5:** Comparison of Small Intestine Length According to Appendix Length Subgroups.

Group	*n*	Mean SIL ± SD (cm)	Median (IQR)	*p*-Value *
Appendix < 8 cm	32	595.6 ± 94.0	573.5 (533.8–637.5)	0.021
Appendix ≥ 8 cm	63	645.4 ± 107.6	625.0 (575.0–720.0)	

Note: Donors with an appendix length ≥ 8 cm had significantly longer small intestines. * Statistical comparison was performed using the Mann–Whitney U test. Abbreviations: SIL: Small Intestine Length; SD: Standard Deviation; IQR: Interquartile Range (q1–q3).

## Data Availability

The data presented in this study are available on request from the corresponding author due to privacy restrictions.
